# Placental Gene Co-expression Network for Maternal Plasma Lipids Revealed Enrichment of Inflammatory Response Pathways

**DOI:** 10.3389/fgene.2021.681095

**Published:** 2021-10-21

**Authors:** Marion Ouidir, Suvo Chatterjee, Pauline Mendola, Cuilin Zhang, Katherine. L. Grantz, Fasil Tekola-Ayele

**Affiliations:** ^1^ Epidemiology Branch, Division of Intramural Population Health Research, Eunice Kennedy Shriver National Institute of Child Health and Human Development, National Institutes of Health, Bethesda, MD, United States; ^2^ Department of Epidemiology and Environmental Health, School of Public Health and Health Professions, University at Buffalo, Buffalo, NY, United States

**Keywords:** pregnancy, gene expression, placenta, cholesterol, LDL-C, co-expression network

## Abstract

Maternal dyslipidemia during pregnancy has been associated with suboptimal fetal growth and increased cardiometabolic diseasse risk in offspring. Altered placental function driven by placental gene expression is a hypothesized mechanism underlying these associations. We tested the relationship between maternal plasma lipid concentrations and placental gene expression. Among 64 pregnant women from the NICHD Fetal Growth Studies–Singleton cohort with maternal first trimester plasma lipids we extracted RNA-Seq on placental samples obtained at birth. Placental gene co-expression networks were validated by regulatory network analysis that integrated transcription factors and gene expression, and genome-wide transcriptome analysis. Network analysis detected 24 gene co-expression modules in placenta, of which one module was correlated with total cholesterol (r = 0.27, P-value = 0.03) and LDL-C (r = 0.31, P-value = 0.01). Genes in the module (n = 39 genes) were enriched in inflammatory response pathways. Out of the 39 genes in the module, three known lipid-related genes (*MPO*, *PGLYRP1* and *LTF*) and *MAGEC2* were validated by the regulatory network analysis, and one known lipid-related gene (*ALX4*) and two germ-cell development-related genes (*MAGEC2* and *LUZP4*) were validated by genome-wide transcriptome analysis. Placental gene expression signatures associated with unfavorable maternal lipid concentrations may be potential pathways underlying later life offspring cardiometabolic traits.

**Clinical Trial Registration:** ClinicalTrials.gov, identifier NCT00912132.

## Introduction

Offspring exposed to maternal dyslipidemia during pregnancy may have suboptimal fetal growth, (Mossayebi et al., 2014; Farias et al., 2017), and may be at higher risk of cardiometabolic diseases in later life such as dyslipidemia, atherosclerosis, hypertension, obesity and type 2 diabetes. (Curhan et al., 1996; Pettitt and Jovanovic, 2001; Samaras et al., 2003). The concept of fetal programming of cardiometabolic diseases is well documented, (Barker, 1998), however the mechanisms are not clearly understood. As the key organ of the maternal-fetal exchange and lipid radicals transfer, the placenta can play a role in this programming. (Myatt, 2006). Maternal dyslipidemia may impact placental growth and function, (Zhang et al., 2017), and has been shown to modify placental epigenome, (Shrestha et al., 2019; Ouidir et al., 2020), where some of these modification may alter gene expression in metabolism-related genes. (Kerr et al., 2018). Studies have reported modifications of placental expression of genes related to lipids in obese compared to lean pregnant women (Hirschmugl et al., 2017) and in rabbits with type-1-diabetes. (Rousseau-Ralliard et al., 2019). Furthermore, some studies have highlighted the impact of a maternal high-fat/obesogenic diet on placental transcriptome among rats (Lin et al., 2019) and mice; (King et al., 2013); however, to our knowledge there has been no study on the impact of maternal lipid concentrations on placental gene expression.

Leveraging differences in gene expression can highlight biological pathways implicated in the influence of maternal dyslipidemia on offspring future health. It is increasingly recognized that the genes that have the greatest difference in expression may not directly drive the phenotype. Therefore, integration of the expression profiles of a group of genes using network analysis (network of correlated gene expressions) could provide novel insights about biological pathways and clinical intervention targets. Gene co-expression networks detect gene modules (groups of co-expressed genes), giving a more robust understanding of the underlying regulatory mechanism. (Hecker et al., 2009). Moreover, genes that are co-expressed are co-regulated by a shared regulatory factor; hence, integrating transcription factors in co-expression networks provides novel insights into shared gene regulatory pathways. (Hartemink et al., 2002). This approach has been successfully used in studies of cancer (Mantini et al., 2020) and obesity (Joseph et al., 2019), but has not been used in lipid-related studies.

Our goal was to investigate the relationship between maternal plasma lipid concentrations (i.e., total cholesterol, high-density lipoprotein cholesterol (HDL-C), low-density lipoprotein cholesterol (LDL-C), and triglycerides) and placental gene co-expression among healthy non-obese pregnant women (Supplementary Figure S1). Specifically, we 1) built placental gene co-expression modules, 2) tested the correlations between the gene co-expression modules and maternal lipid traits, and 3) validated our findings using integrative network analysis and genome-wide gene-expression analysis.

## Materials and Methods

### Study Population

This study involved 64 pregnant women from the *Eunice Kennedy Shriver* National Institute of Child Health and Human Development (NICHD) Fetal Growth Studies–Singleton cohort who had plasma lipid concentrations measured at enrollment and provided placenta samples at delivery from which RNA was extracted. The overall cohort included 2,334 non-obese pregnant women enrolled between 8 weeks and 6 days and 13 weeks and 6 days between July 2009 and January 2013 from 12 clinic sites within the US. (Grewal et al., 2018) To be enrolled, women had to have no past adverse pregnancy outcomes or self-reported behavioral risk factors such as use of cigarettes, illicit drugs or alcohol in the months prior to pregnancy. The study was approved by institutional review boards at NICHD, each participating clinical site and data coordinating centers. This study has been performed in accordance with the principles of the Declaration of Helsinki.

### Maternal Blood Lipid Concentration Measurements

Total cholesterol, HDL-C and triglyceride concentrations in plasma were measured using maternal non-fasting blood samples collected at enrollment (10 weeks 0 days–13 weeks 6 days of pregnancy). Lipid measurement methods have been previously published. (Shrestha et al., 2019; Ouidir et al., 2020). Briefly, LDL-C was calculated using the Friedewald formula, (Friedewald et al., 1972), while other lipids were directly measured using the Roche COBAS 6000 chemistry analyzer (Roche Diagnostics, Indianapolis, IN). The inter-assay laboratory coefficients of variation were 2.2, 3.2, and 2.3% for total cholesterol, HDL-C and triglycerides, respectively. Lipids were dichotomized using clinically accepted cut-points for cardiovascular disease risk comparing unfavorable versus favorable values based on the third report of the National Cholesterol Education Program (NCEP III) criteria (Expert Panel on Detection and Adults, 2001): high vs normal total cholesterol (≥200 mg/ dl vs < 200 mg/ dl), low *vs*. normal HDL-C (≤50 mg/ dl *vs*. > 50 mg/ dl), high vs normal LDL-C (≥100 mg/dl *vs*. < 100 mg/ dl), and high vs normal triglycerides (≥150 mg/dl *vs*. < 150 mg/ dl).

#### Placenta RNA Quantification for Gene Expression

Within 1 h after delivery, trained personnel rinsed the placenta with sterile saline, pat dried, removed nonadherent bloods clots, trimmed placental membrane and umbilical cord and collected placenta biopsies measuring 0.5 cm × 0.5 cm x 0.5 cm directly below the fetal surface. (Tekola-Ayele et al., 2020). Samples were placed in RNALater and frozen. Processing was performed at the Columbia University Irving Medical Center; RNA was extracted from biopsies (*n* = 80) using TRIZOL reagent (Invitrogen, MA), and sequenced using the Illumina HiSeq2000 system. (Delahaye et al., 2018). The expression of the transcripts were quantified using Salmon (Patro et al., 2017) which accounts for experimental attributes and biases such as fragment GC-content bias that is commonly observed in RNA-seq data; 19,087 protein-coding genes were available for analysis. A total of 64 participants had both placental RNA-seq and maternal lipid concentration data.

#### Statistical Analysis

##### Gene Co-expression Network Construction

To construct gene co-expression modules, we performed a gene co-expression network analysis (Zhang and Horvath, 2005) using the Weighted Gene Co-expression Network Analysis (WGCNA) R package. (Langfelder and Horvath, 2008). To reduce noise in the co-expression network, 436 genes with missing gene expression values in more than 95% samples or with zero variance were excluded, leaving 18,651 protein-coding genes for analysis. Then, as recommended by authors of the WGCNA package, we have done a variance-stabilizing transformation using the “varianceStabilizingTransformation” function from the DESeq2 R package. (Love et al., 2014). To create the gene co-expression modules we chose the soft power of 6, which was the lowest power for which the scale-free topology fit index curve reached a saturation point at a high value (R^2^ > 0.8) and a satisfying mean summary connectivity (Supplementary Figure S2). We performed a one-step network construction and module detection with a soft-threshold power of 6, an “unsigned” topological overlap matrix, a minimum module size of 30 and a merge cut height = 0.25 (i.e, the branches of the hierarchical clusters were cut at height of 0.25 to define modules). The hierarchical clustering dendrogram was successfully generated (Supplementary Figure S3). Next, for each gene module, we computed its eigengene (first principal component of the expression matrix).

##### Correlations Between Lipids and Co-expression Modules

The correlations between lipid concentrations in the overall samples as well as stratified by lipid cut-points (i.e., high vs normal total cholesterol, low *vs*. normal HDL-C, high *vs.* normal LDL-C, and high *vs*. normal triglycerides) and eigengene of each co-expression module were tested using the “cor” function as implemented in the WGCNA package. (Langfelder and Horvath, 2008). For each gene belonging to a module that was significantly correlated with a lipid trait, we calculated module membership and gene significance as implemented in WGCNA. Module membership estimates the degree of correlation between gene expression and module eigengene (calculated using the first principal component of the expression matrix of the module) i.e., the module membership quantifies the correlation of the gene expression with the module. Gene significance indicates the correlation between gene expression and the lipid trait. The higher the correlation between module membership and gene significance, the more reliable is the association between the module and the lipid trait. In order to identify the gene that best explains the functional features of the module, we identified the hub gene (i.e., the gene with the highest connectivity: high module membership and high gene significance). (Bhuva et al., 2019). Further, we assessed whether placental expression of the hub genes differed between women with favorable *vs*. unfavorable lipid profiles.

##### Canonical Pathway Analysis

All genes belonging to a co-expression module that was significantly correlated with maternal clinically unfavorable lipid concentrations were further explored for canonical pathways, networks, and diseases and biological function using the “Core Analysis” function in Ingenuity Pathway Analysis (IPA, QIAGEN, Redwood City, CA, United States, www.qiagen.com/ingenuity). Statistically significant overrepresented canonical pathways were determined by Fisher’s exact test followed by adjustment for multiple testing using the Benjamini-Hochberg method.

##### Gene Regulatory Network Analysis

While co-expression networks have been successful, they do not explicitly integrate the biological mechanisms involved in regulating gene expression, such as the binding of transcription factors (TFs). We implemented an approach known as Passing Attributes between Networks for Data Assimilation **(**PANDA) (Glass et al., 2013) with three inputs: our placental gene expression data, a TF motif prior and a set of known protein-protein interactions (PPI) from NetZooR package. To create specific transcriptional regulatory networks for each lipid trait, we ran PANDA using the same TF motif prior and PPI data, but separately including gene expression from women with clinically unfavorable vs favorable lipid concentrations for each lipid trait. For each lipid trait, we calculated edge weight (the degree of connection between TF and target gene) in dyslipidemic women and edge weight in non-dyslipidemic women. We then identified subnetworks by selecting high-probability edges specific to clinically unfavorable lipid profiles, using one probability combining the probabilities that an edge is both “supported” and “different”, as described in Glass et al. (Glass et al., 2015) We select edges for which this combined probability is greater than 97%; this cutoff was chosen so that each subnetwork contains roughly 0.05% of all possible edges obtained in our analysis (*n* = 653,729) in each group. We verified the robustness of our network analysis to this cutoff by varying it systematically between 80 and 99% (Supplementary Figure S4), for which similar results were found (Supplementary Figure S5).

##### Genome-wide Associations

As a validation method, we tested genome-wide associations between mRNA levels of protein-coding genes and each maternal lipid profiles (i.e., high total cholesterol *vs*. normal, high LDL-C *vs*. normal, high triglycerides *vs*. normal and low HDL-C *vs*. normal) using the R/Bioconductor package DESeq2. (Love et al., 2014). DESeq2 implements negative binomial generalized linear models and estimates dispersion and logarithmic fold changes to quantify differential expression. Adjustment factors in the model included maternal race/ethnicity, maternal age in years, fetal sex, and the first 10 genotype principal components. The Benjamini-Hochberg adjusted *p*-value of the Wald test was used to correct for multiple testing.

##### Comparison Between the Different Methods Used

The three analyses (i.e., co-expression modules analysis, gene regulatory network analysis and the genome-wide associations) were performed separately, and the last two were used as validation of the co-expression modules analysis. The results of the analysis have been compared looking at the overlaps of genes in the co-expression modules and the differentially expressed genes from the network analysis and the genome-wide analysis. Overlaps using the co-expressed modules significantly associated with maternal lipids levels were presented in Venn-diagram using the “vennDiagram” function from the R/Bioconductor package limma, (Ritchie et al., 2015), while overlaps with non-significant modules were presented in a Table as percentage of the number of genes by module.

## Results

Among the 64 women included in the analysis the mean (standard deviation) maternal age and pre-pregnancy BMI were 27.6 (5.8) yeas and 23.3 (3.0) kg/ m^2^, respectively, and 22% had high total cholesterol, 44% had high LDL-C, 17% had high triglycerides and 16% had low HDL-C at enrollment (Table 1). There was no significant difference in characteristics of women included in our analytic sample and the full NICHD Fetal Growth Study cohort (Table 1). The co-expression network construction resulted in 24 placental gene co-expression modules. The size of the modules ranged from 31 genes (module “darkturquoise”) to 5,923 genes (module “turquoise”), with a median size of 534 genes per module (Supplementary Table S1).

**TABLE 1:** Characteristics of the study subsample (*n* = 64) compared to the remaining samples not included in the present study from the NICHD fetal Growth Studies–Singletons (total *n* = 2,334).

Characteristics	Participants included (*n* = 64)	Participants not included (*n* = 2,270)	*p*-value
	Mean ± sd or N (%)	Mean ± sd or N (%)	
**Maternal age, years**	27.6 ± 5.8	28.2 ± 5.5	0.37
**Maternal pre-pregnancy BMI, kg/m^2^**	23.3 ± 3.0	23.3 ± 3.1	0.33
**Maternal race/ethnicity**			0.30
Non-Hispanic white	18 (28.1)	596 (26.3)	
Non-Hispanic black	17 (26.6)	594 (26.2)	
Hispanic	22 (34.4)	627 (27.6)	
Asian and Pacific Islander	7 (10.9)	453 (20.0)	
**Gestational age at enrollment, weeks**	12.7 ± 1.0	12.7 ± 1.0	0.85
**Gestational age at delivery, weeks**	39.2 ± 1.2	39.2 ± 1.8	0.81
**Fetal sex**			0.96
Male	33 (51.6)	1072 (47.2)	
Female	31 (48.4)	994 (43.8)	
**Maternal clinically unfavorable lipid trait**			
High total cholesterol	14 (21.9)	659 (29.0)	0.18
High LDL-C	28 (43.8)	999 (44.0)	0.80
High triglycerides	11 (17.2)	514 (22.6)	0.25
Low HDL-C	10 (15.6)	437 (19.3)	0.41

BMI, body mass index; LDL-C: low-density lipoprotein cholesterol; HDL-C: high-density lipoprotein cholesterol.

### Total Cholesterol and LDL-C are Related to Placental Expression of Genes Implicated in Inflammatory Response

**FIGURE 1:**
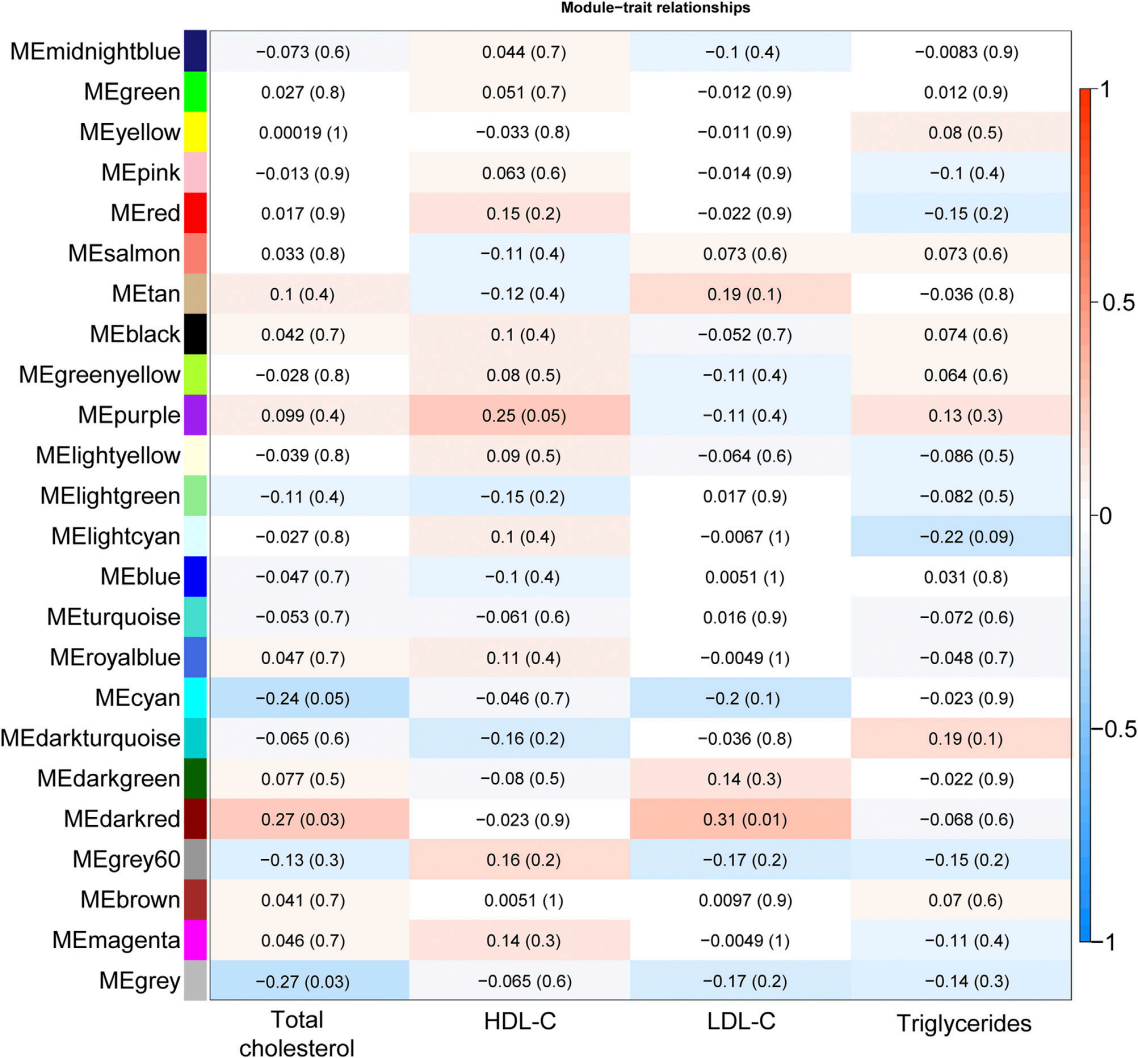
Heatmap plot illustrating the correlation of module-lipid trait relationships for the detection of the gene modules mostly associated with lipid traits. Each element in the heatmap represents the Pearson correlation (and P-value) between module eigengenes (y-axis; calculated using the first principal component of the gene expression matrix of each module) and lipid trait (x-axis); the color gradient represents the strength of the correlation.

**FIGURE 2:**
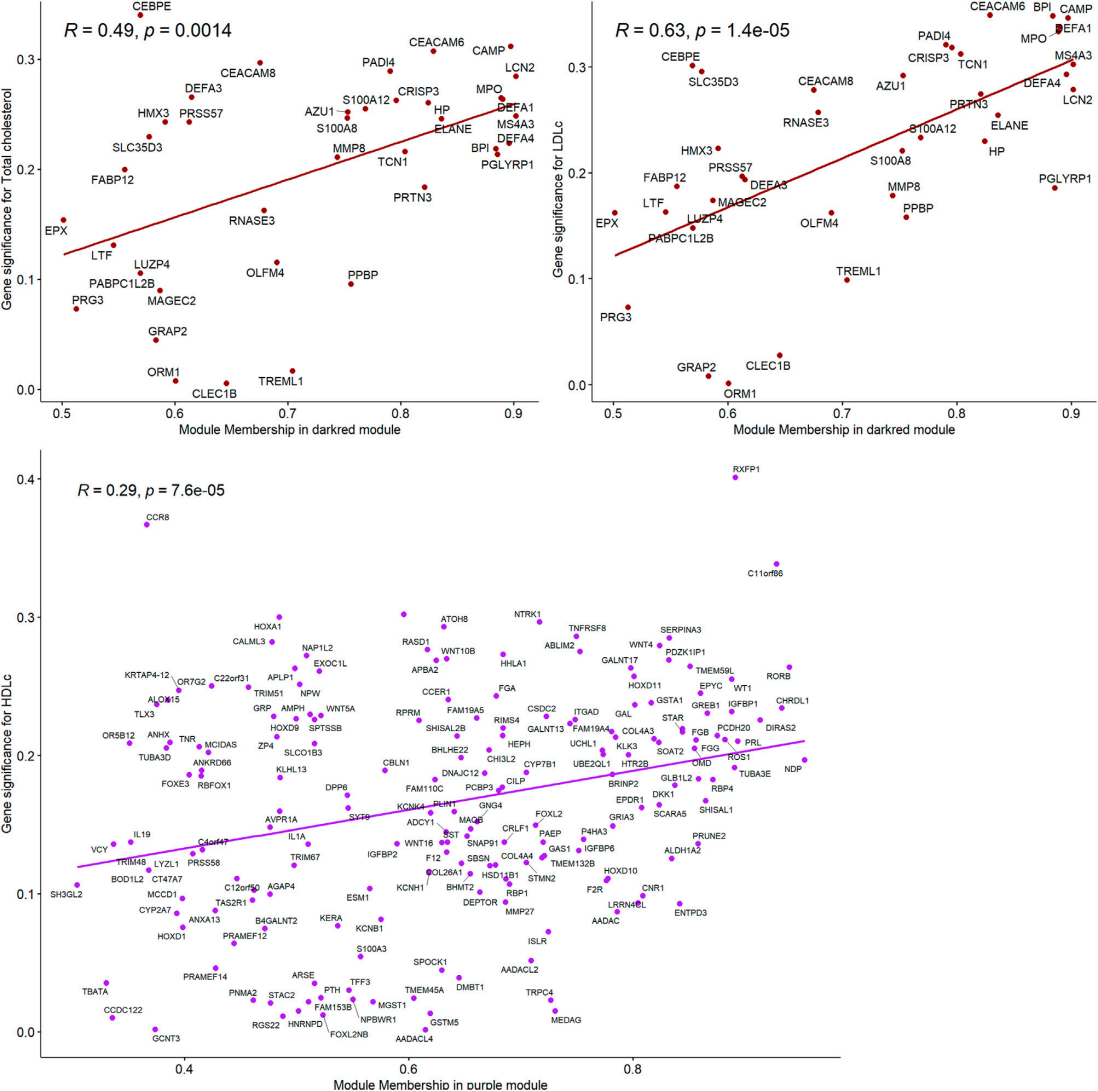
Correlations between gene module memberships and gene significance for the module “darkred” and Total cholesterol and LDL-C and for the module “purple” and HDL-C.

Total cholesterol and LDL-C were significantly correlated with the “darkred” module (r = 0.27, P-value = 0.03 and r = 0.31, P-value = 0.01, respectively, Figure 1), composed of 39 placental co-expressed genes of which *LCN2* is the hub gene (Supplementary Table S2). *LCN2* gene expression in placenta did not differ by total cholesterol status (P-value = 0.93) but tended to be upregulated in women with high LDL-C status (P-value = 0.085, Supplementary Figure S6). The correlation between “darkred” module and lipid concentrations was stronger in the group of women with unfavorable lipid concentrations than favorable lipid concentrations (r = 0.29, p-value = 0.02 *vs*. r = 0.19, p-value = 0.13 for high *vs.* normal total cholesterol and r = 0.29, p-value = 0.02 *vs*. r = 0.00, *p*-value = 0.99 for high *vs*. normal LDL-C, Table 2). “Darkred” module membership was highly correlated with gene significance for total cholesterol (r = 0.49, *p*-value = 0.001) and gene significance for LDL-C (r = 0.63, p-value = 1.4 × 10^−5^), confirming that the association between the module and the two lipid traits was highly reliable (Figure 2). Protein-protein interactions in the “darkred” module are presented in the Supplementary Figure S7A

**TABLE 2:** Pearson correlation (and p-value) between module eigengenes (from modules associated with lipid traits) and lipid concentrations where lipid traits were divided in clinically favorable *vs*. unfavorable lipid concentration groups (i.e., high total cholesterol, high LDL-C, low HDL-C).

Lipid trait	Module	Favorable lipid concentration group		Unfavorable lipid-concentration group	
		r	p-value	R	p-value
Total cholesterol	darkred	0.192	0.128	0.293	0.019
LDL-C	darkred	0.000	0.999	0.290	0.020
HDL-C	purple	0.251	0.045	0.366	0.003

LDL-C, low-density lipoprotein cholesterol; HDL-C, high-density lipoprotein cholesterol.

NOTE: Triglycerides is absent from the table because it was not associated with any gene co-expression module.

The genes included in the “darkred” module were enriched in canonical IPA disease and function pathways mostly related to inflammatory response and disease (FDR *p*-values ranged from 1.14 × 10^−2^ to 1.64 × 10^−31^; Supplementary Table S3). The top IPA canonical pathways included pathways related to inflammation and cardiometabolic function such as IL-8 Signaling (*AZU1*, *DEFA1*, *MPO*; *p*-value = 0.004) that plays a central role in angiogenesis, tumor growth and inflammation; liver X receptor/retinoid X receptor (LXR/RXR) Activation (*ORM1, S100A8*; p-value = 0.018) which is involved in the regulation of lipid metabolism, inflammation, and cholesterol; and Atherosclerosis Signaling (*ORM1*, *S100A8*; p-value = 0.019; Supplementary Table S4). The top IPA canonical networks were enriched in inflammatory response, cardiovascular disease and cardiovascular system development and function (Supplementary Table S5).

**FIGURE 3:**
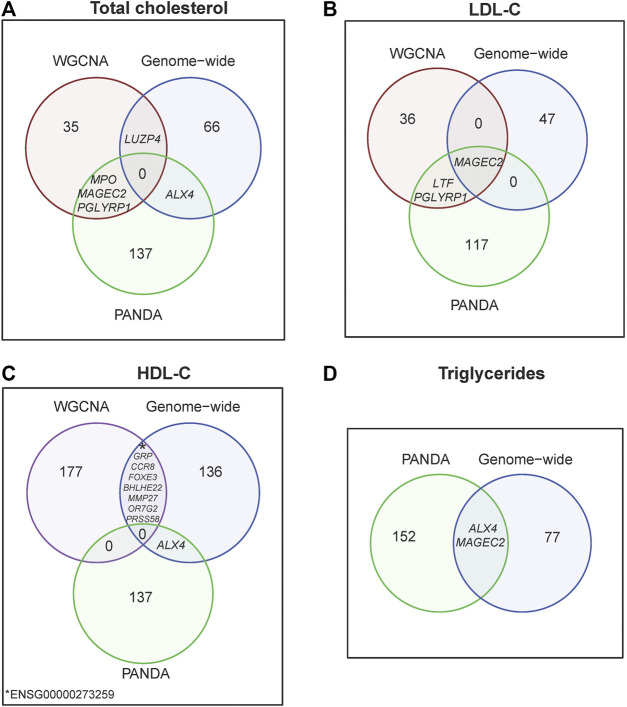
Venn diagram by lipid traits **(A)** Total cholesterol, **(B)** LDL-C, **(C)** HDL-C and **(D)** Triglycerides, illustrating top-differentially expressed genes from WGNCA, PANDA and genome-wide analyses.

Further validation analyses found that four placental co-expressed genes in the “darkred” module (*MAGEC2*, *PGLYP1*, *LUZP4* and *MPO*) overlapped with a set of 141 genes that were associated with high total cholesterol based on regulatory network analysis (Supplementary Figure S8A; Supplementary Table S6) or 68 genes that were associated with high total cholesterol based on genome-wide gene expression analysis (Supplementary Table S7; Figure 3A, Supplementary Figure S9A). Likewise, three placental co-expressed genes in the “darkred” module (*MAGEC2*, *PGLYP1*, and *LFT*) overlapped with a set of 120 genes found to be associated with LDL-C based on regulatory network analysis (Supplementary Figure S8B; Supplementary Table S6) or 48 genes found to be associated with high LDL-C based on genome-wide gene expression analysis (Figure 3B; Supplementary Figure S9B). Overlaps with all the co-expression gene modules showed highest overlap percentage with the “darkred” module for total cholesterol and second highest for LDL-C and triglycerides (Supplementary Table S8).

### HDL-C Is Marginally Correlated With One Gene Co-expression Module

HDL-C was marginally correlated with the “purple” module composed of 185 placental co-expressed genes (r = 0.25, p-value = 0.05, Figure 1), characterized by the hub gene *NDP*. *NDP* was significantly downregulated in placenta of women with low HDL-C status (p-value = 0.001, Supplementary Figure S6C). The correlation between “purple” module and lipid concentrations was stronger in the group of women with unfavorable HDL-C concentrations than favorable HDL-C concentrations (r = 0.37, p-value = 0.003 *vs*. r = 0.25, p-value = 0.05 for low vs normal HDL-C, Table 2). “Purple” module membership was correlated with gene significance for HDL-C (r = 0.29, p-value = 6.7 × 10^−5^, Figure 2). Protein-protein interactions in the “purple” module are presented in the Supplementary Figure 7B

Further validation analyses found that eight placental co-expressed genes in the “purple” module (*BHLHE22*, *CCR8*, *FOXE3*, *GRP*, *MMP27*, *OR7G2*, *PRSS57* and *ENSG00000273259*) overlapped with a set of 145 genes found to be associated with low HDL-C based on genome-wide gene-expression analysis (Figure 3C, Supplementary Figure S9C). All differentially expressed genes significantly associated with total cholesterol overlapped with genes significantly associated with HDL-C (Supplementary Figure S10).

### Triglycerides Are Not Associated With Any Gene Co-expression Module

Triglycerides were not significantly associated with any gene co-expression module. However, *ALX4* and *MAGEC2* were significantly associated with triglycerides based on both gene regulatory network analysis (that found 154 triglyceride-associated genes, Supplementary Figure S8D) and genome-wide gene-expression analysis (that found 79 triglyceride-associated genes, Supplementary Table S7; Figure 3D).

## Discussion

Using gene co-expression approach validated by genome-wide analysis approaches, we identified placental gene regulatory networks significantly associated with unfavorable maternal lipid concentrations in early pregnancy. There was a considerable convergence of the lipids-associated regulatory networks, genes, and enriched pathways at loci relevant to lipid metabolism and transportation, cardiovascular disease risk, and inflammatory response. A notable finding of our study was the consistent associations of total cholesterol and LDL-C with the “darkred” module, characterized by the hub gene *LCN2* (Lipocalin 2). *LCN2* plays a role in transportation of small hydrophobic molecules such as lipids, steroid hormones and retinoids; furthermore *LCN2* is a candidate cardiovascular disease gene and may function as a modulator of inflammation. (Flo et al., 2004).

The three genes in the “darkred” module associated with total cholesterol and LDL-C in both gene co-expression and regulatory network analyses (*LTF*, *MPO* and *PGLYRP1*) are well-known lipid related genes. *LTF* *(*lactotransferrin) is a major iron-binding protein highly expressed in lactating breast, and its protein product has been associated with decreased serum triglycerides concentration in mice and rats. (Takeuchi et al., 2004; Tamano et al., 2008). In humans, circulating level of lactoferrin has been positively associated with HDL-C and negatively associated with triglycerides, BMI, waist-to-hip ratio and fasting glucose. (Moreno-Navarrete et al., 2008). Moreover, variants in *LTF h*ave been associated with triglycerides and HDL-C concentrations. (Moreno-Navarrete et al., 2008). *MPO* (myeloperoxidase) is a heme protein that plays a role in the oxidative modification of lipoproteins. Serum myeloperoxidase contributes significantly to HDL-C functionality. (Variji et al., 2019). In a previous genome-wide association study (GWAS), *MPO* has been associated with plasma biomarkers of cardiovascular risk. (Folkersen et al., 2017). *PGLYRP1* (peptidoglycan recognition protein 1) has been associated with cardiovascular risk factors such as diabetes, hypertension, higher concentration of total cholesterol, lower concentration of HDL-C and history of myocardial infarction. (Rohatgi et al., 2009).

Additional three genes (*MAGEC2*, *LUZP4*, *ALX4*) from the “darkred” module associated with total cholesterol and LDL-C in gene co-expression analysis were validated by the genome-wide analysis. *MAGEC2* (MAGE family member C2) and *LUZP4* (leucine zipper protein 4) are expressed normally only in immune privileged sites (testis or placenta), and their restricted expression suggests that they may function in germ cell development. (Djureinovic et al., 2016). *ALX4* (ALX homeobox 4) is essential for embryonic morphogenesis, (Bertola et al., 2013), and previous GWASs have found variants in *ALX4* associated with blood pressure (He et al., 2013) and LDL-C. (Drenos et al., 2009). We have previously found that high maternal triglycerides are associated with decreased methylation of a CpG site in *ALX4*. (Ouidir et al., 2020).

Pathway analysis revealed placental inflammation response to unfavorable maternal lipid concentrations including LXR/RXR activation, where RXR is known to interact with peroxisome proliferator-activated receptors (PPARs, α, β/δ, and γ) where PPAR family plays a major regulatory role in energy homeostasis and metabolic function by reducing the triglyceride concentration, increasing insulin sensitization and enhancing fatty acid metabolism. (Tyagi et al., 2011). Many studies have identified LXR and PPARs as sensors of fatty acids and lipids and mediators of their effects on gene expression. (Yoshikawa et al., 2002; Salter and Tarling, 2007). Furthermore, PPAR is highly expressed in the placenta and has a crucial role in placenta development. Canonical pathway analysis of the co-expressed genes associated with total cholesterol and LDL-C also reported atherosclerosis signaling response in the placenta while previous studies have reported higher offspring risk of progressive atherosclerosis associated with maternal hypercholesterolemia. (Palinski et al., 2007; Nasioudis et al., 2019).

We acknowledge that our study’s sample size is too small to detect co-expression networks that may have a modest correlation with lipid concentrations. However, even with a modest sample size, we identified significant association at loci relevant to lipid metabolism with a considerable convergence of the different analyses. Our criteria for unfavorable lipid profiles are from a non-pregnant population and lipid concentrations were limited to sampling in the first trimester. Lipids are known to rise dramatically in pregnancy with substantial changes in blood lipid concentrations after the first trimester of pregnancy for some women. (Grantz et al., 2019). Our study provides important insight into the understanding that maternal lipid concentration as early as the first trimester were associated with placental gene expression at delivery. Future studies with longitudinal lipid sampling are needed to further elucidate this relationship. Furthermore, we cannot conclude whether the gene expression changes are a cause or consequence of unfavorable maternal lipid profiles because there are gestational age-dependent changes in pregnant women’s blood lipid concentrations (Grantz et al., 2019) and placental gene expression profiles. (Tarrade et al., 2015). Our study has several strengths: this is the first study of placental gene co-expression in relation to maternal lipid concentrations. The study cohort comprised non-obese healthy pregnant women (i.e. without history of adverse pregnancy outcomes and behavioral risk factors such as use of cigarettes, illicit drugs or alcohol in the months prior to pregnancy), minimizing potential confounding. We were able to validate our findings using three approaches: co-expression network focusing on concordant changes in gene expression, integrative networks leveraging conditional regulatory relationships between transcription factors and gene expression (i.e. identifying changes in regulation of different gene expression across women with unfavorable/favorable lipid profiles), and genome-wide analysis facilitating detection of differential expression at specific genes.

Our study provided corroborating and coherent evidence related to the association between maternal lipids and placental gene co-expression. However, our study was limited in providing mechanistic insights, where further experimental studies are needed. Specifically, the hub gene *LCN2* and genes that were validated across different analyses approaches such as *MAGEC2*, *LUZP4*, *ALX4*, *MCUB*, *LTF*, *MPO* and *PGLYRP1* can be taken forward by further experimental work to identify molecular regulatory processes and further understand the mechanism by which maternal lipid levels may modify the placental gene expression.

## Conclusion

In summary, we found that early pregnancy unfavorable lipid concentrations based on common clinical cut-points were significantly associated with placental gene expression at loci relevant to lipid metabolism, transportation, and inflammatory response. This study highlighted the potential role of the hub gene *LCN2* known for its role in lipids transportation and potential inflammatory modulation. The findings provide novel insight about the potential role of placental gene expression mechanisms in mediating the relationship between unfavorable maternal lipid profiles and fetal development as well as future risk of offspring cardiovascular diseases.

## Data Availability

Publicly available datasets were analyzed in this study. This data can be found here: the datasets analyzed for this study can be found in the dbGaP with accession number phs001717. v1. p1 (https://www.ncbi.nlm.nih.gov/projects/gap/cgi-bin/study.cgi?study_id = phs001717.v1.p1). The maternal genotype data analyzed in the current study are available from the corresponding author upon request.
