# Supplementary material for: Placental Gene Co-expression Network for Maternal Plasma Lipids Revealed Enrichment of Inflammatory Response Pathways
Source: Front Genet. 2021 Oct 21;12:681095. doi: 10.3389/fgene.2021.681095 (PMC8567461; doi:10.3389/fgene.2021.681095)

**Placental gene co-expression network for maternal plasma lipids revealed enrichment of inflammatory response pathways**

Marion Ouidir^1^, Suvo Chatterjee^1^, Pauline Mendola^1,2^, Cuilin Zhang^1^, Katherine L. Grantz^1^, Fasil Tekola-Ayele^1^.

^1^ Epidemiology Branch, Division of Intramural Population Health Research, *Eunice Kennedy Shriver* National Institute of Child Health and Human Development, National Institutes of Health, Bethesda, MD, USA

^2^ Department of Epidemiology and Environmental Health, School of Public Health and Health Professions, University at Buffalo, Buffalo, NY, USA

**SUPPLEMENTARY FIGURES**

**Supplementary** **Figure 1**: **Illustration of the analysis workflow** (example using maternal plasma total cholesterol concentration in early pregnancy and placental gene expression) First, we performed a gene co-expression network analysis using the Weighted Gene Co-expression Network Analysis (WGCNA) to identify a module with significant Pearson’s correlation with a lipid trait. Second, we identified regulatory relationships of transcription factors and gene expression using Passing Attributes between Networks for Data Assimilation **(**PANDA). Finally, we performed a genome-wide transcriptome analysis as a validation to detect individual genes associated with maternal lipid trait.

**
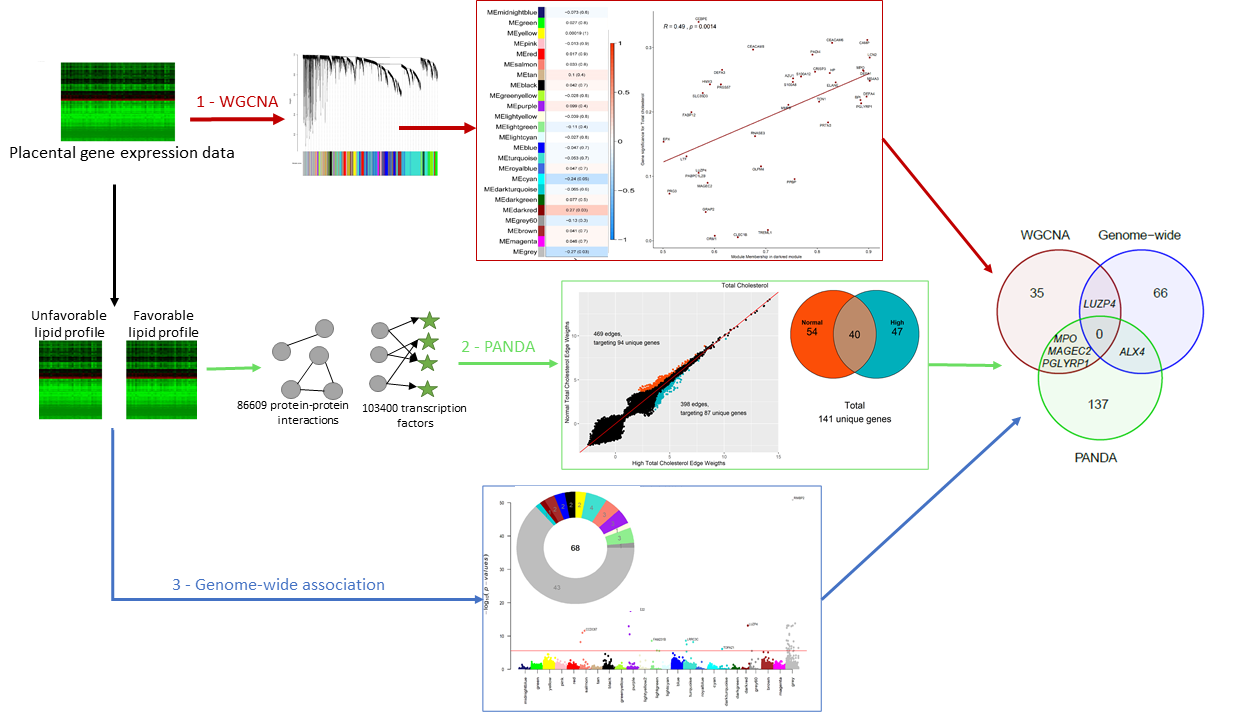
**

**Supplementary** **Figure 2**: Network properties for different soft thresholds. Points are labeled by the corresponding adjacency function parameter. The horizontal red line represents the 0.80 scale-free topology (R^2^).


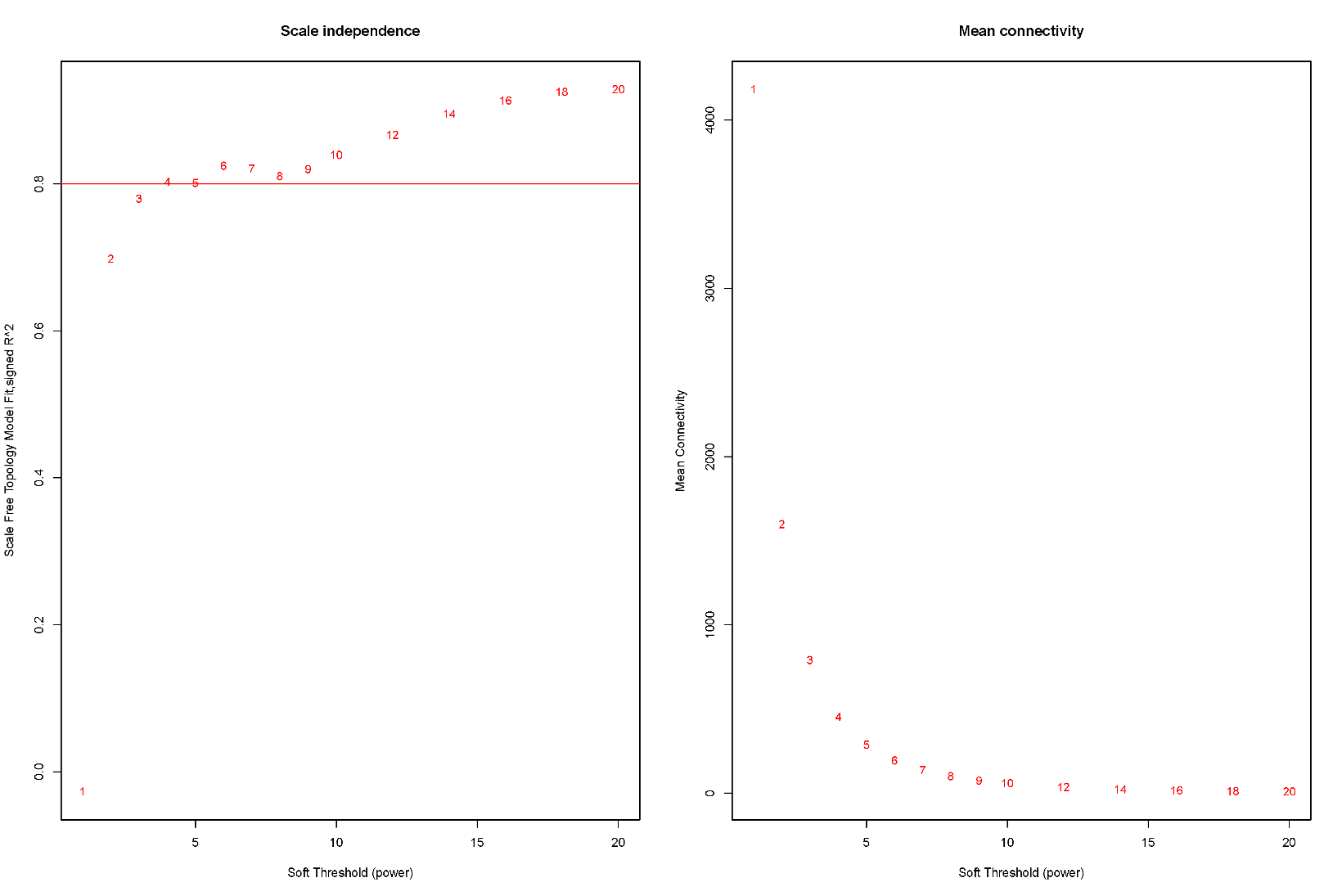


**Supplementary** **Figure 3**: Cluster dendrogram of 18651 protein-coding genes of the human placenta co-expression network. The color bands show module assignment of genes based on a one-step automatic block analysis.


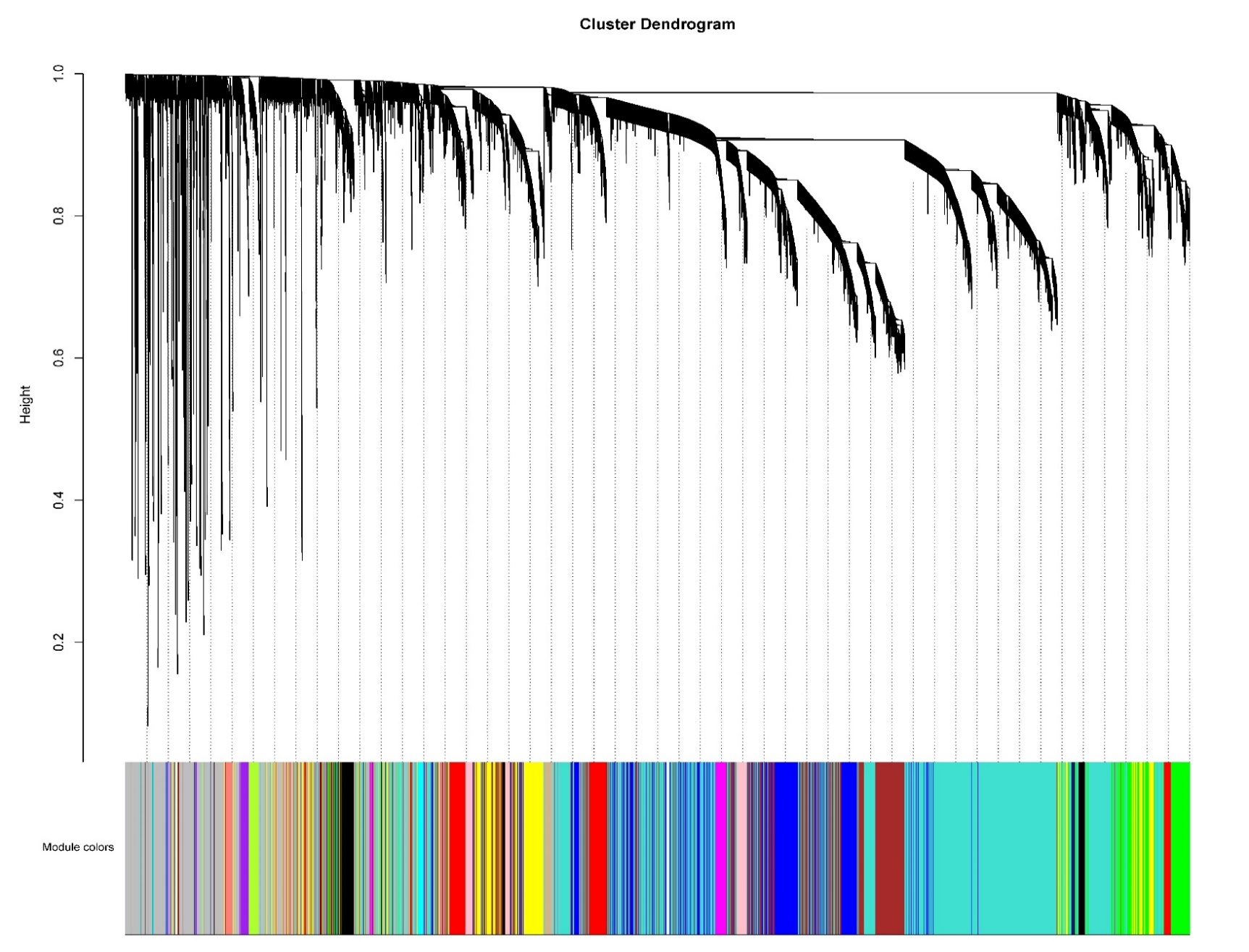


**Supplementary** **Figure 4**: Impact of selected high-probability edges cutoff to identify subnetworks after the Passing Attributes between Networks for Data Assimilation **(**PANDA) analysis. We tested all cutoff between 80% and 99% by increment of 1 for each maternal lipid trait.


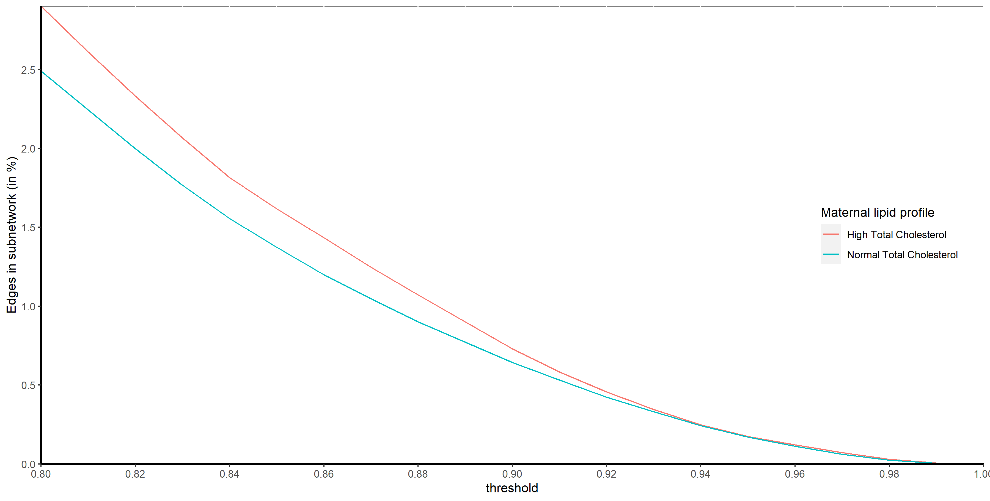

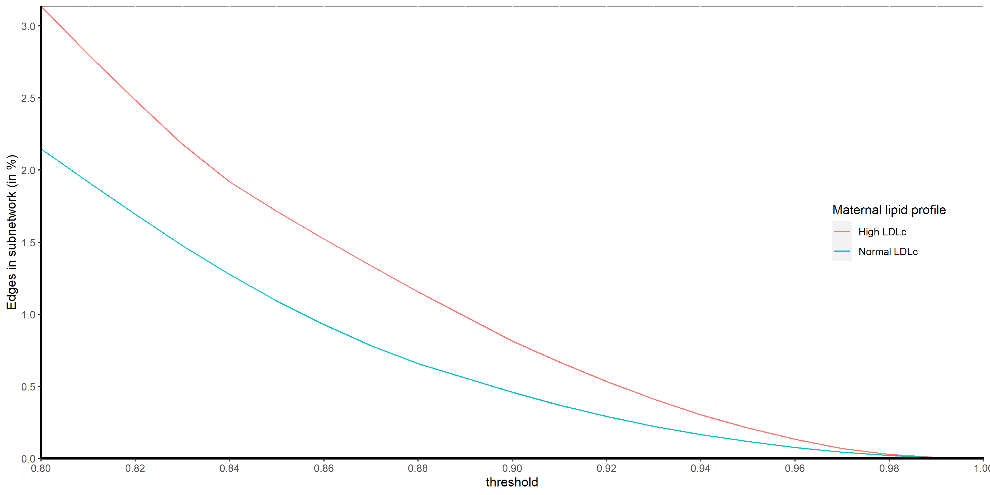

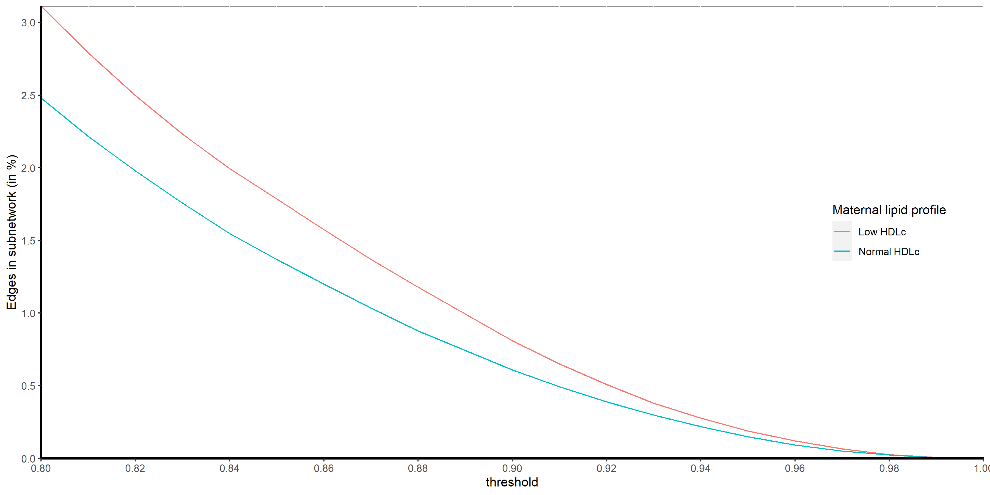

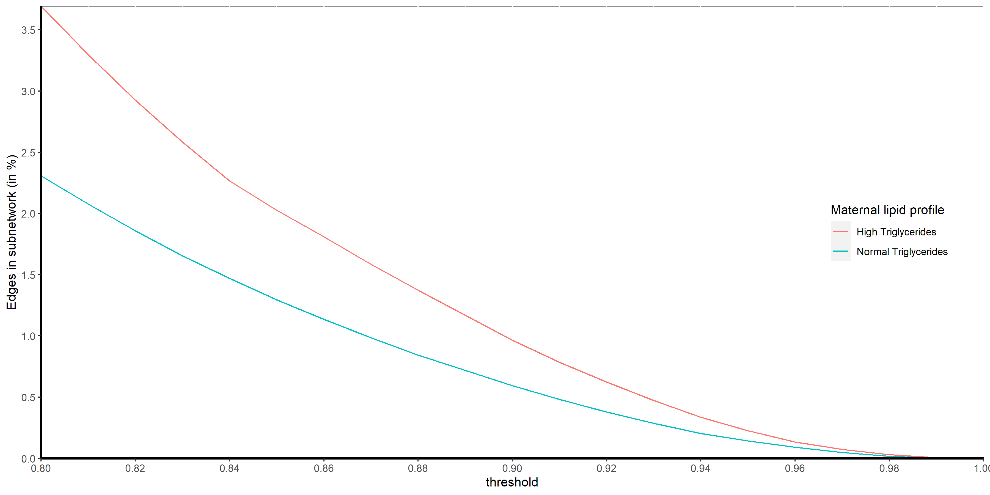


**Supplementary Figure 5**: Impact of selected high-probability edges cutoff to identify subnetworks after the Passing Attributes between Networks for Data Assimilation **(**PANDA) analysis and comparison with genes significant in the WGCNA ‘darkred’ module and the genes significantly differentially expressed with total cholesterol.


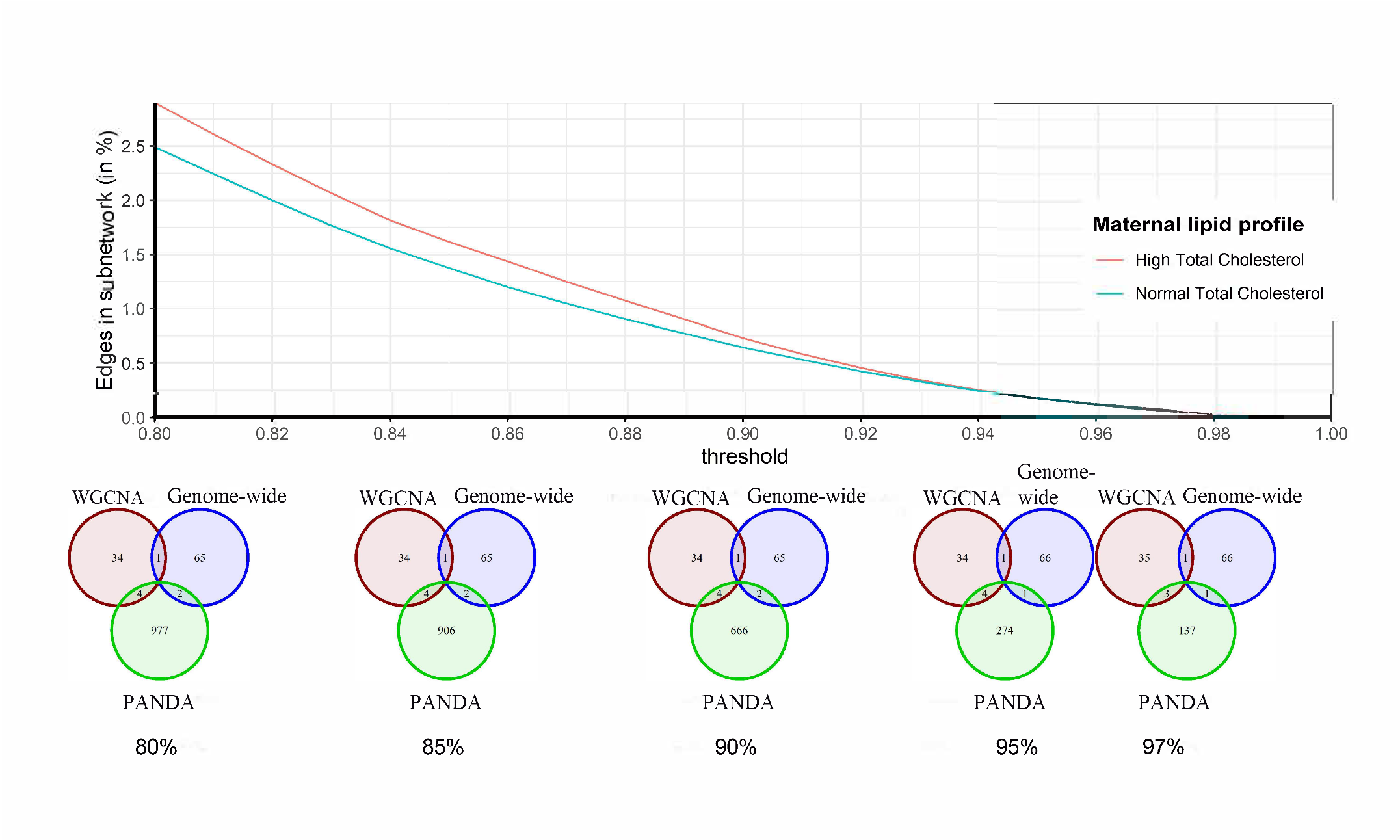


**Supplementary Figure 6:** Placental expression of the hub genes between women with favorable vs. unfavorable lipid profiles **(A)** *LCN2* gene expression by cholesterol status; **(B)** *LCN2* gene expression by LDL-C status and **(C)** *NDP* gene expression by HDL-C status.

**
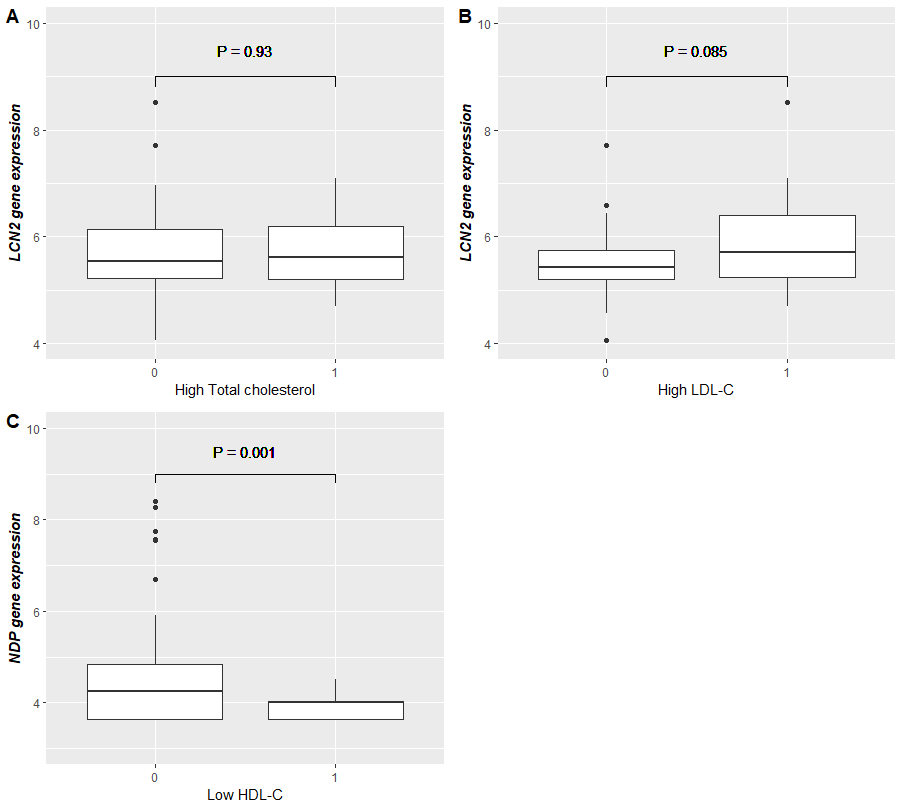
**

**Supplementary** **Figure 7**: Protein-protein interactions in lipids-associated modules. Each node denotes a gene and each line indicates a protein-protein interaction. (A) Protein-protein interactions in the “darkred” module. (B) Protein-protein interactions in the “purple” module.

A - Module “darkred” (PPI enrichment p-value:< 1.0e-16)


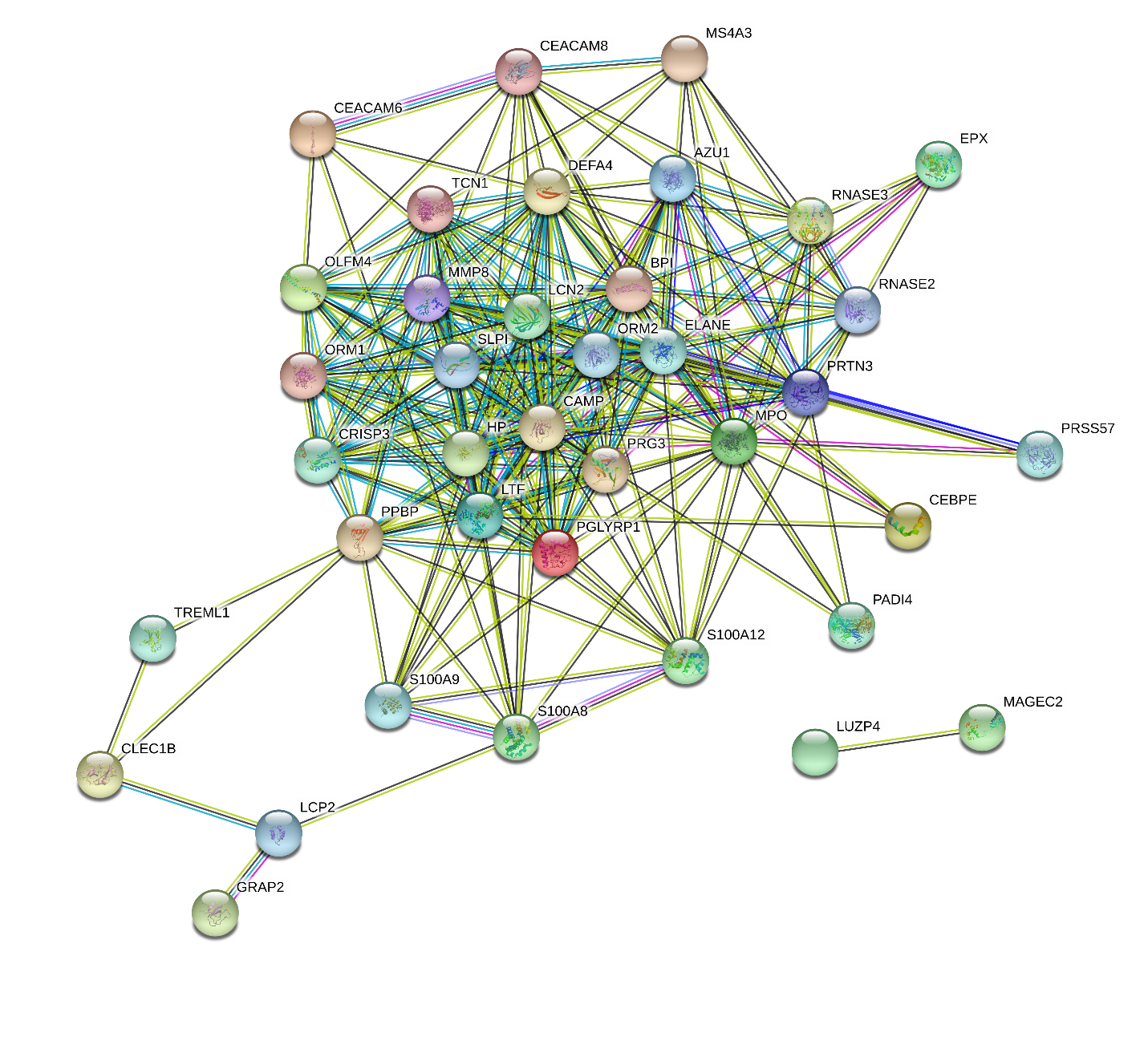


B - Module “purple” (204 edges, PPI enrichment p-value:< 1.0e-16)


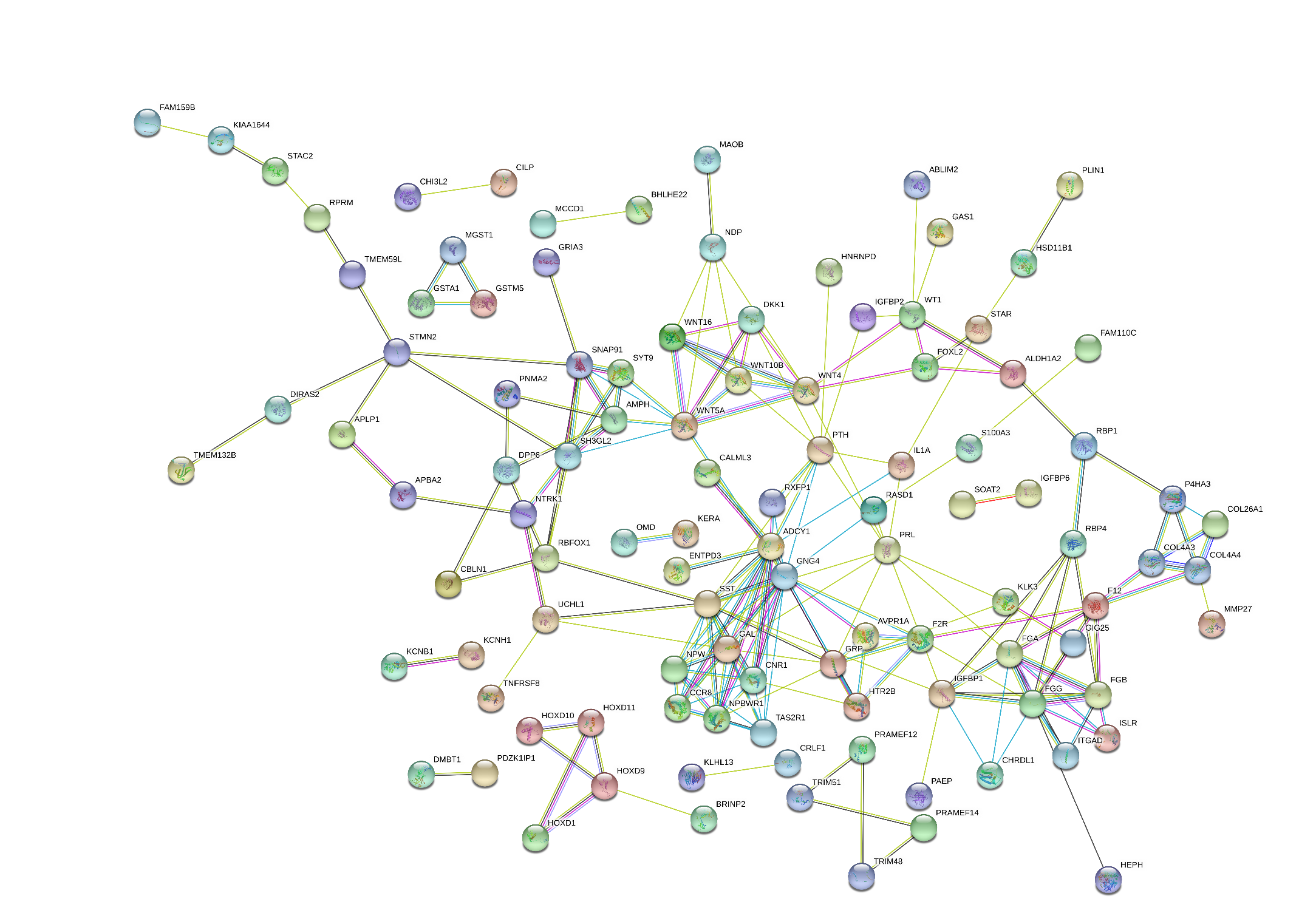


**
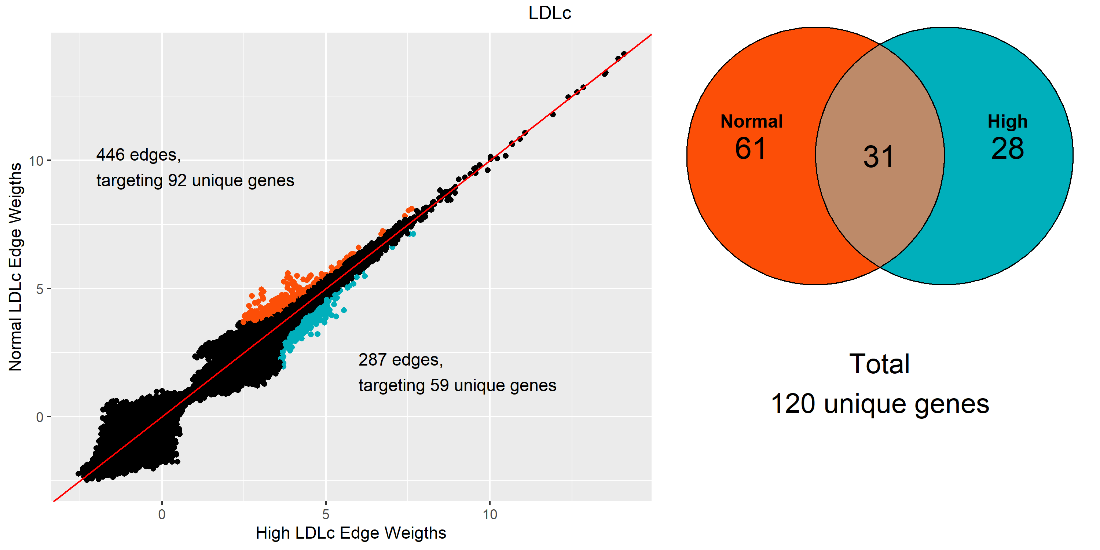

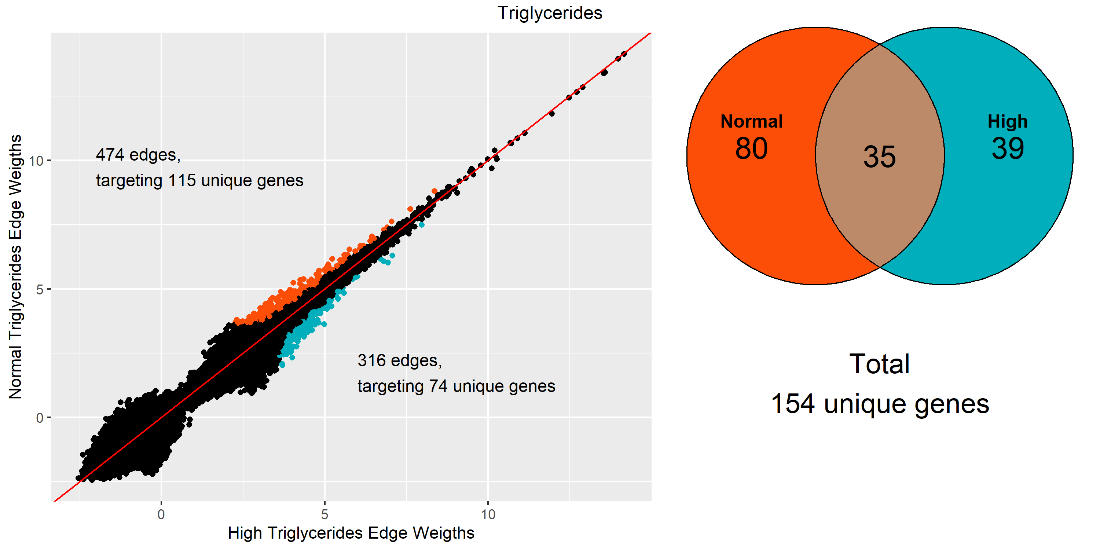

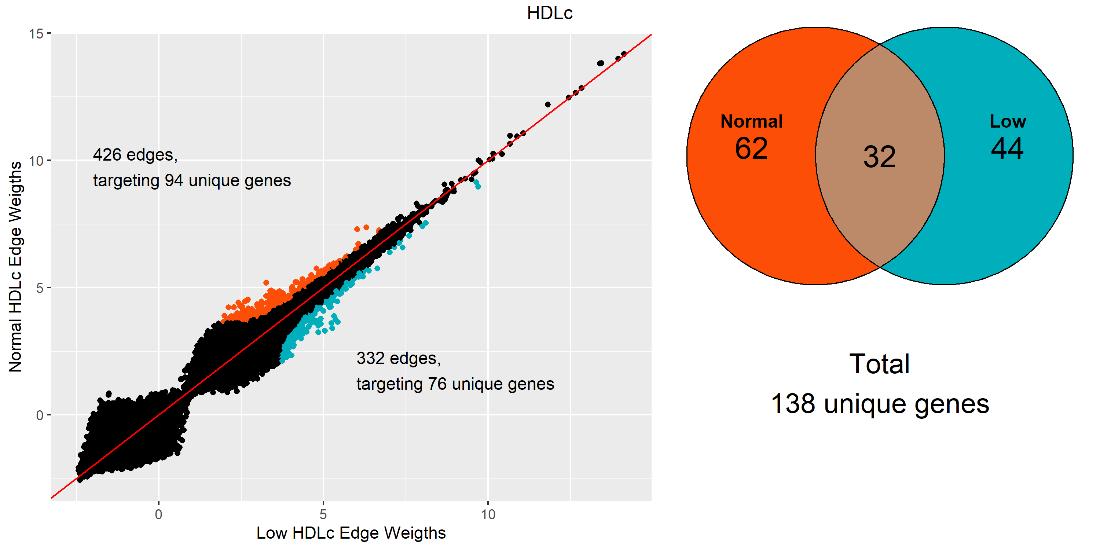

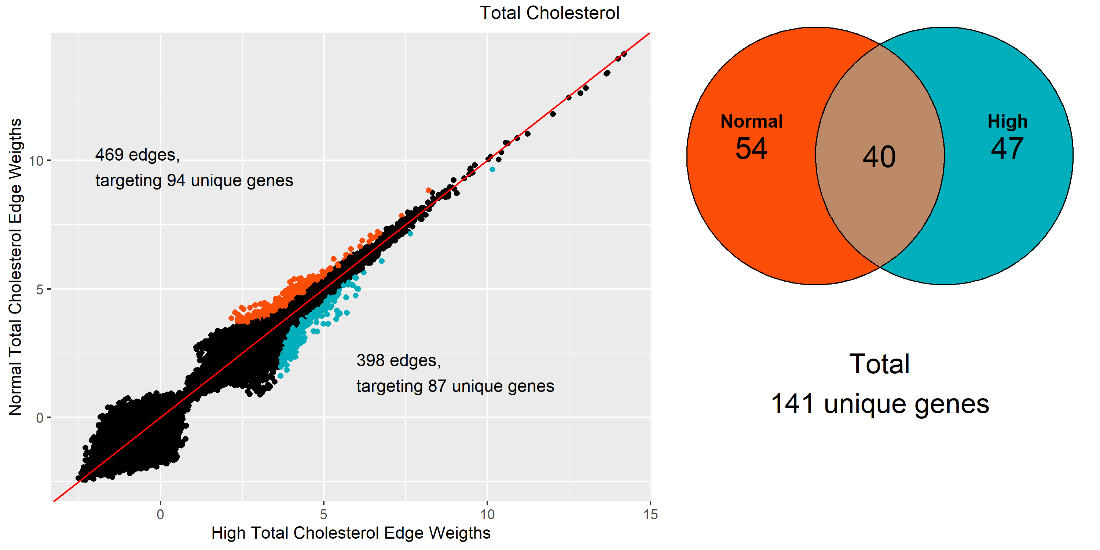
Supplementary** **Figure 8**: PANDA edge difference by lipids trait **(A)** Total cholesterol, **(B)** LDL-C, **(C)** HDL-C and **(D)** Triglycerides. Each point in the graph represents an edge connecting a TF to a target gene. We also define subnetworks by selecting high-probability edges (>97%) specific to either the favorable lipid profile (red) and unfavorable lipid profile (blue) model. The numbers of edges and unique genes identified as part of these subnetworks are noted.

**Supplementary Figure 9**: Manhattan plots associations between placental gene expression and maternal lipid profiles for **(A)** Total cholesterol, **(B)** LDL-C, **(C)** HDL-C and **(D)** Triglycerides.


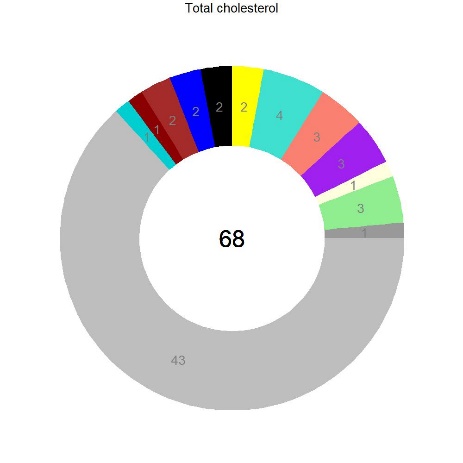

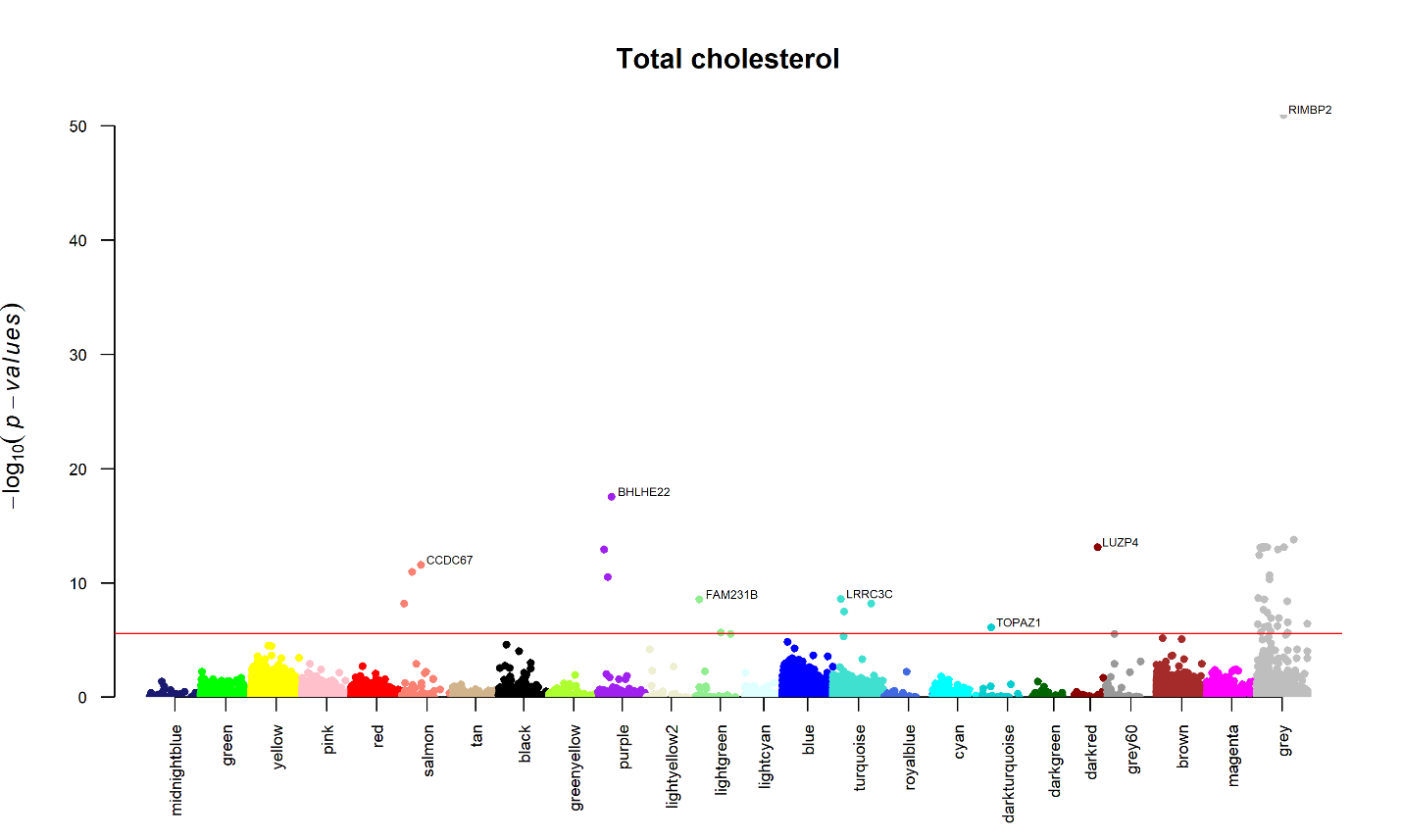


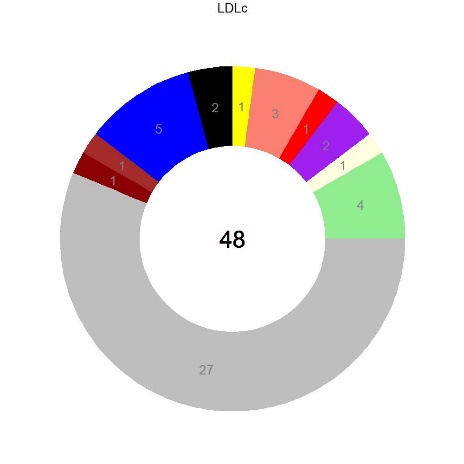

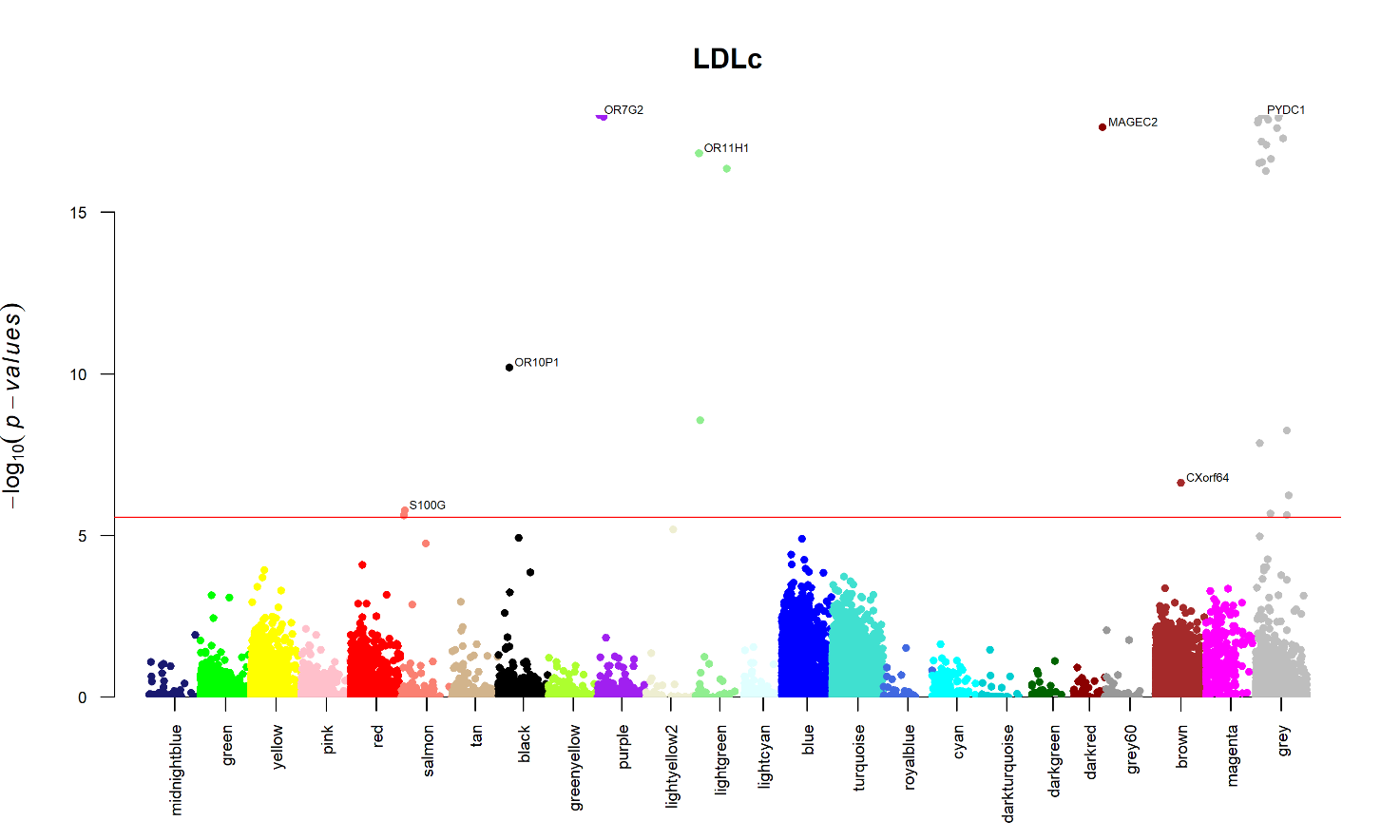


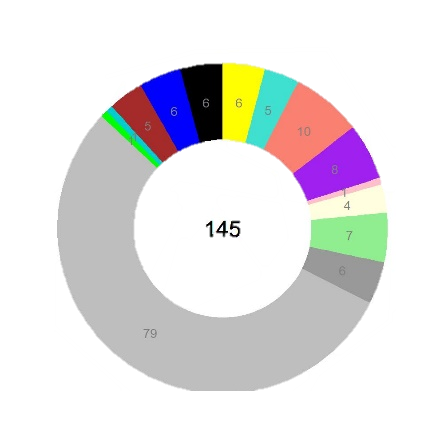

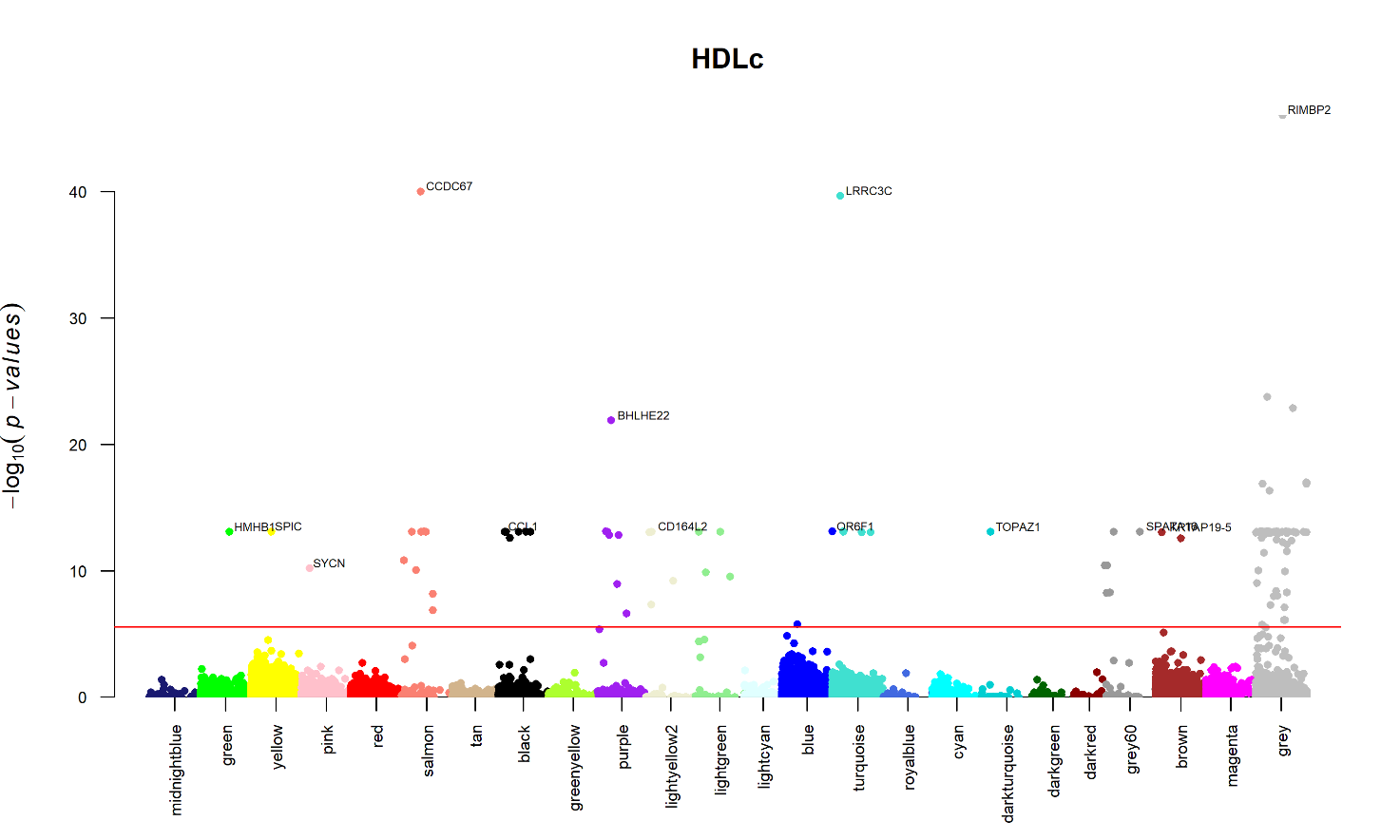


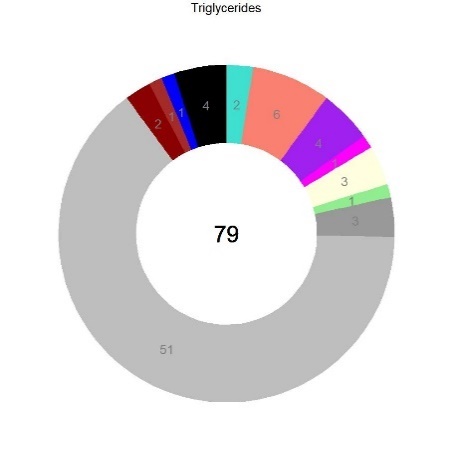

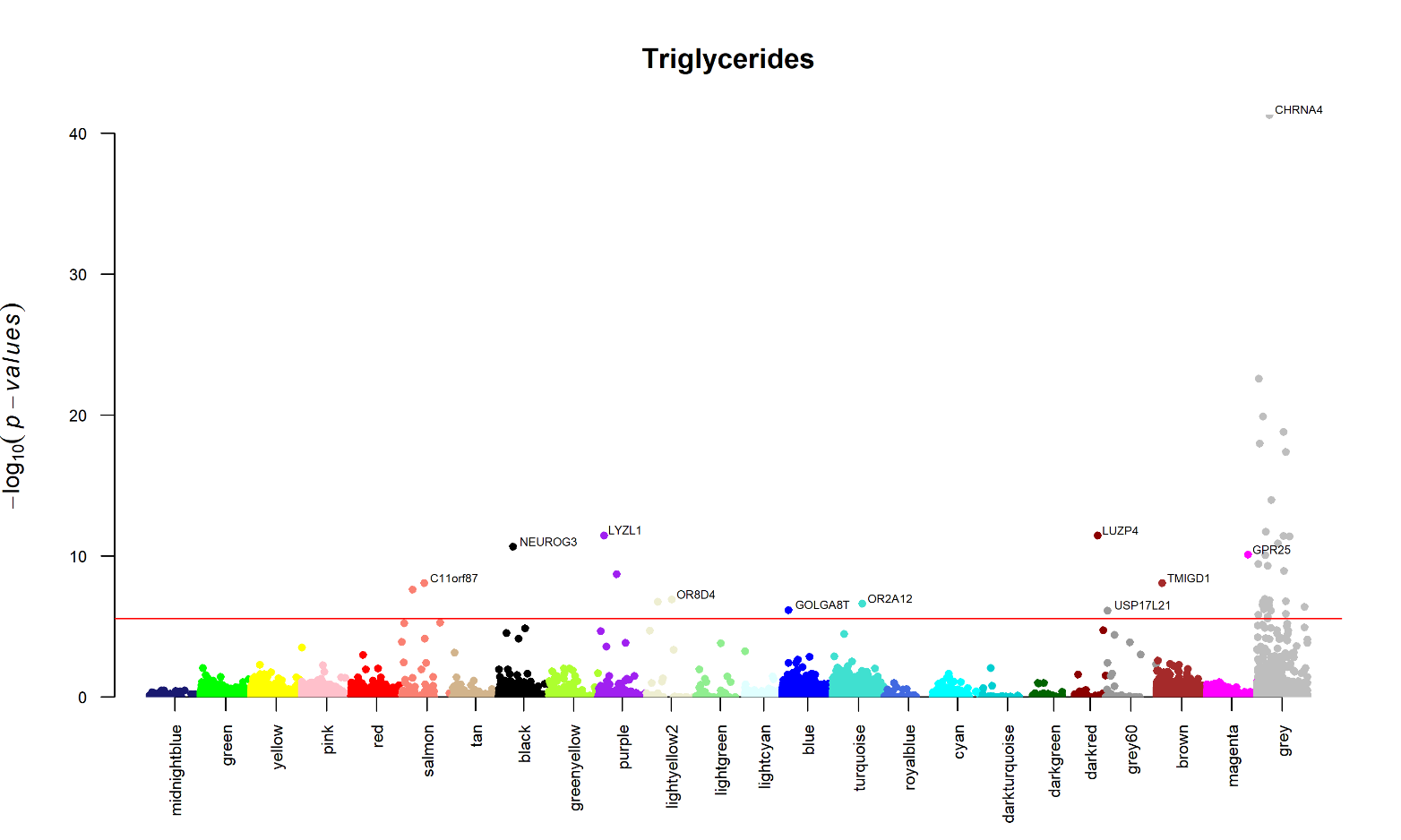


**Supplementary** **Figure 10**: Area-Proportional Euler diagram on genome-wide results.


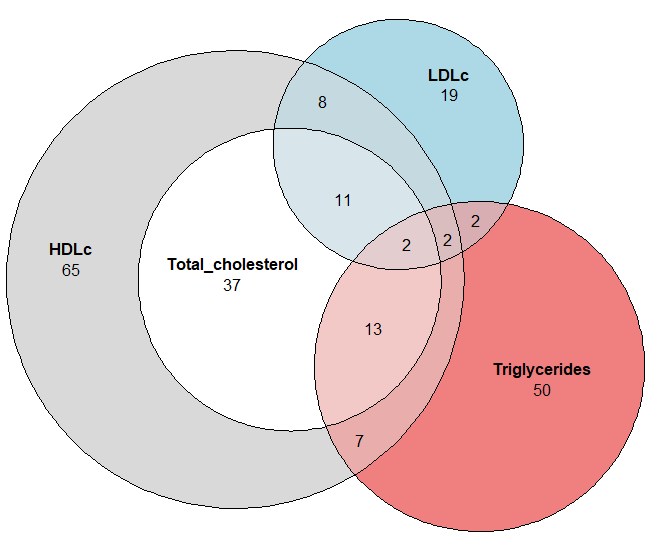

Supplement: Supplementary file 1 [file DataSheet2.docx]
